# Phase Evolution of Li-Rich Layered Li-Mn-Ni-(Al)-O Cathode Materials upon Heat Treatments in Air

**DOI:** 10.3390/ma17246056

**Published:** 2024-12-11

**Authors:** Jekabs Grins, Aleksander Jaworski, Leif Olav Jøsang, Jordi Jacas Biendicho, Gunnar Svensson

**Affiliations:** 1 Arrhenius Laboratory, Department of Materials and Environmental Chemistry, Stockholm University, SE-10691 Stockholm, Sweden; jekabs.grins@mmk.su.se (J.G.); aleksander.jaworski@mmk.su.se (A.J.); 2 Cerpotech, Kvenildmyra 6, 7093 Heimdal, Norway; leifolav.josang@cerpotech.com; 3 Catalonia Institute for Energy Research-IREC, Sant Adrià de Besòs, 08930 Barcelona, Spain; jjacas@irec.cat

**Keywords:** Li-Mn-Ni-O, Li-rich layered oxides, XRPD, NPD, phase evolution

## Abstract

The phase evolution of Li-rich Li-Mn-Ni-(Al)-O cathode materials upon heat treatments in the air at 900 °C was studied by X-ray and neutron powder diffraction. In addition, the structures of Li_1.26_Mn_0.61−x_Al_x_ Ni_0.15_O_2_, x = 0.0, 0.05, and 0.10, were refined from neutron powder diffraction data. For two-phase mixtures containing a monoclinic Li_2_MnO_3_ type phase M and a rhombohedral LiMn_0.5_Ni_0.5_O_2_ type phase R, the structures, compositions, and phase fractions change with heat treatment time. This is realized by the substitution mechanism 3Ni^2+^ ↔ 2Li^+^ + 1Mn^4+^, which enables cation transport between the phases. A whole-powder pattern fitting analysis of size and strain broadening shows that strain broadening dominates. The X-ray domain size increases with heat treatment time and is larger than the sizes of the domains of M and R observed by electron microscopy. For heat-treated samples, the domain size is smaller for R than for M and decreases with increasing Al doping.

## 1. Introduction

Heat treatment experiments were carried out throughout more comprehensive studies of cobalt-free Li-rich cathode materials with nominal compositions Li_1.1_Mn_0.55−x_Al_x_Ni_0.35_O_2_ [1,2], Ni35, and Li_1.26_Mn_0.61−x_Al_x_Ni_0.15_O_2_ [3], Ni15, within the EU project COBRA [4]. The Al content was varied between x = 0, Al00, and x = 0.10, Al10.

Al doping is an effective strategy to enhance the electro-chemical performance of layered oxides used as cathodes in Li-ion batteries [5]. An example is LiNi_0.8_Co_0.15_Al_0.05_O_2_ (NCA), a commercially available cathode for high-energy cells. Its long cycle life has been correlated to the small amounts of Al in the layered structure, diminishing transition metal dissolution and mitigating irreversible phase transformations on cycling [6]. Similar beneficial effects have been reported for Al doping in Li-rich layered oxides; it reduces voltage fade while improving capacity retention from 68% to 96% after 100 cycles for Li_1.2_Ni_0.16_Mn_0.56_Co_0.08_O_2_ [7]. In Li-rich Co-free oxides with the formula Li_1.26_Mn_0.61_Ni_0.15_O_2_, Al doping promotes the formation of a core–shell structure, stabilizes the cathode, and mitigates spinel phase transformation on cycling. This results in an enhanced capacity retention of 67% compared to 46% for the undoped phase after 250 cycles [3].

The materials were characterized by techniques including X-ray and neutron powder diffraction (XRPD and NPD), Raman spectroscopy, and nuclear magnetic resonance (NMR), and their electro-chemical performance was determined. Phase distributions within grains were studied using scanning transmission electron microscopy (STEM) and data from selected area electron diffraction (SAED), electron energy loss spectroscopy (EELS), and energy-dispersive X-ray analysis (EDX). A statistical methodology, denoted hypermodal data fusion (HyDF), was used to analyze correlations between the different data types and to extract common spectral features in the STEM data (“phases”) [8,9].

**Figure 1:**
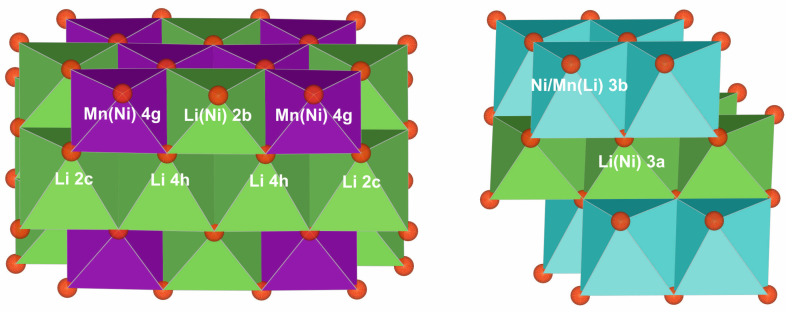
Octahedral illustrations of the monoclinic M phase (**left**) and rhombohedral R phase (**right**). The multiplicity and Wyckoff letter for the sites are indicated.

Two main phases were observed; a monoclinic phase M with *C*2/*m* symmetry and ideal composition Li(Li_1/3_Mn_2/3_)O_2_ ≡ Li_2_MnO_3_ and a rhombohedral LiMn_1/2_Ni_1/2_O_3_ type phase R with *R*-3*m* symmetry; Mn and Ni ideally present in both phases as Mn^4+^ and Ni^2+^. The two structure types are illustrated in Figure 1 (cf. also Figure S1). In the M phase, Ni may be incorporated into the 4g and 2b sites. In the R phase, the transition metal layer may contain Li and the Li layer may contain Ni. The phases have similar structures and often similar compositions. At high d values, there are a few isolated characteristic super-structure reflections from M, but these are weak and their shapes are affected by the presence of stacking faults, making them difficult to use in refinements, cf. 3.1 below. They are, therefore, often difficult to distinguish in XRPD patterns [2]. In the case of severe peak overlap, the main reflections from M and R do have different structure factors, but it is, nevertheless, sometimes not practically feasible to refine both their structures from XRPD data, while that still may be possible using NPD data [2]. Their individual presence may, of course, be established by using TEM.

The parent Al-free compositions lie approximately between Li_2_MnO_3_ and LiMn_1/2_Ni_1/2_O_2_, and may formally be written as a combination of these, (1 − 2x)Li(Li_1/3_Mn_2/3_)O_2_ − 2xLiMn_1/2_Ni_1/2_O_2_, e.g., with x = 0.35 for Ni35.

The Ni35 materials were found to be two-phase mixtures of M and R [2], with Ni incorporated into M according to 3Ni^2+^ ↔ 2Li^+^ + 1Mn^4+^ [10]. One Ni substitutes for one Mn on the 4 g site and two Ni substitutes for two Li on the 2b site, while the 4h and 2c sites remain occupied by only Li. The compositions of the Al-free M and R phases were derived to be ~Li_1.26_Mn_0.63_Ni_0.11_O_2_ and ~Li_0.88_Mn_0.44_Ni_0.68_O_2_. The Ni15 materials are here by NPD found to be single-phase M. These findings agree with those reported by McCalla [11,12], who found two corners of a three-phase region to be Li_1.22_Mn_0.62_Ni_0.16_O_2_ and Li_0.8_Mn_0.34_Ni_0.86_O_2_ for quenched samples prepared at 800 °C in O_2_ atmosphere.

For the Al-doped Ni35 materials [2], it could not be reliably concluded into which phase(s) Al enters and it was assumed in [2] that Al enters M and R in equal proportions. The nominal formulas used in this study for both Ni35 and Ni15 are not strictly charge-balanced. Charge balance may be realized in many ways, one possibility being by a reduction followed by an expulsion of Li_2_O, which can transform into carbonate in air. The materials were by NPD and NMR found to contain minor amounts of Li_2_CO_3_. For Ni35, the amounts are small, less than ~1 wt%, justifying the use of the nominal compositions in the diffraction data analysis [2]. For Ni15, the amounts are larger, e.g., for Al10 2.4 wt%, corresponding to ~Li_1.26_Mn_0.51_Al_0.10_Ni_0.15_O_2_ × 0.026Li_2_CO_3_, indicating that their true M compositions may contain less Li than nominally.

We present here studies of how the M and R phases change when tempered at 900 °C in two-phase mixtures of M + R (Ni35) and single-phase M (Ni15). The compositions of the phases are found to vary considerably with the holding time at 900 °C. The study is, thus, motivated by the hope that it could provide knowledge for the syntheses of these and similar materials. Short-term heat treatments of Ni35-Al01 were made to investigate the influence of calcination time. Longer-term heat treatments of Al-doped Ni35 were made on pressed pellets to investigate the evolvement towards equilibrium. The studies are largely based on the accurate determinations of unit cell parameters and the whole-powder pattern fitting analysis of size and strain broadening.

In addition, the structures of single-phase M Ni15-Al00/Al05/Al10 were refined from NPD data in order to establish how similar these structures are to the M-phases found in two-phase M + R Ni35 samples.

## 2. Materials and Methods

**Figure 2:**
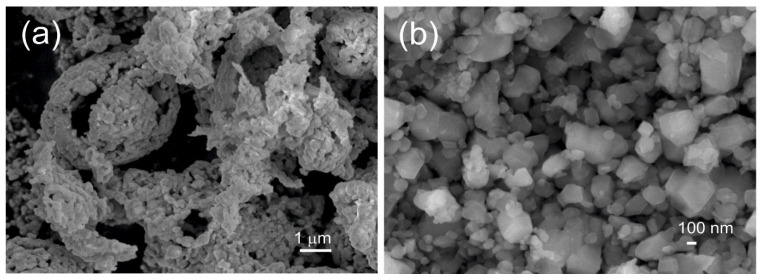
Secondary electron images of Ni35-Al01: (**a**) calcined at 900 °C for 6 h and (**b**) after milling.

The materials were synthesized by aqueous precursor-based spray pyrolysis. The constituting metal nitrates were dissolved in distilled water and mixed in stoichiometric amounts. The solution was atomized using a two-phase nozzle (pressurized air + solution) into a preheated rotating (~2 rpm) furnace (Entech Energiteknik AB) under constant air flow with an approximate average residence time of ~2 s at 900 °C. The powders were then calcined at 900 °C for 6 h in a dry air atmosphere, consisting of partly agglomerated grains with an average grain size of ~0.1 μm, as shown in Figure 2b.

**Figure 3:**
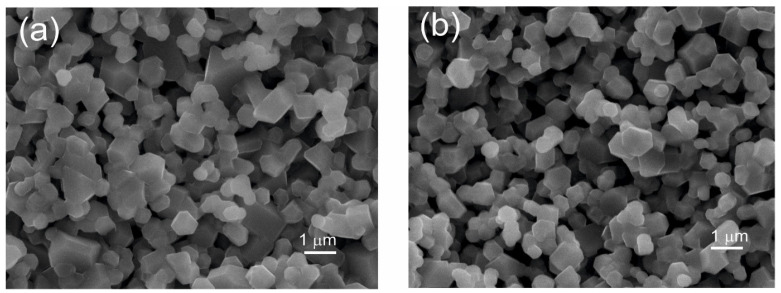
Secondary electron images of Al-doped Ni35 samples heat-treated at 900 °C for 3 weeks: (**a**) Ni35-Al05; (**b**) Ni35-Al10.

The heat treatment experiments were performed in the air using a Mo-silicide element heated muffle furnace from Entech Energiteknik, AB, Ängelholm, Sweden. Short-term heat treatments were carried out on powders, in order to replicate synthesis conditions, in alumina crucibles, and long-term heat treatments on pressed pellets in order to facilitate material transfer. The furnace was heated at 5°/min to 900 °C, held there for various times, and then turned off and the furnace hatch opened. The temperature dropped to 700 °C within minutes and to 85 °C within half an hour. Considerable grain growth is observed after heat treatment at 900 °C for 3 weeks, as shown in Figure 3, with an average grain size of ~1 μm. The corresponding cross section polished surfaces are shown in Supplementary Materials Section S2.1.

Scanning electron microscopy (SEM) images were recorded with a JSM-7000F microscope, JEOL Ltd., Tokyo, Japan. NPD data for Ni15 Li_1.26_Mn_0.61−x_Al_x_Ni_0.15_O_2_, x = 0, 0.05, and 0.10, were collected with the D2B diffractometer at Institute Laue-Langevin (ILL), France, with a wave-length of 1.5406 Å. XRPD data were collected as previously specified [3] (cf. also Supplementary Materials Section S2.2) using NIST 640c Si as an internal standard for 2θ and to approximate the instrumental peak broadening. Structure refinements were made with the FullProf software, version 8.10 [13] (cf. S2.3 and S2.4). Errors for the derived domain sizes and strains were calculated according to the expressions given by Bhakar et al. [14] (cf. S2.5).

## 3. Results

### 3.1. Heat Treatments of Ni35-Al01 at 900 °C for Short Times

**Figure 4:**
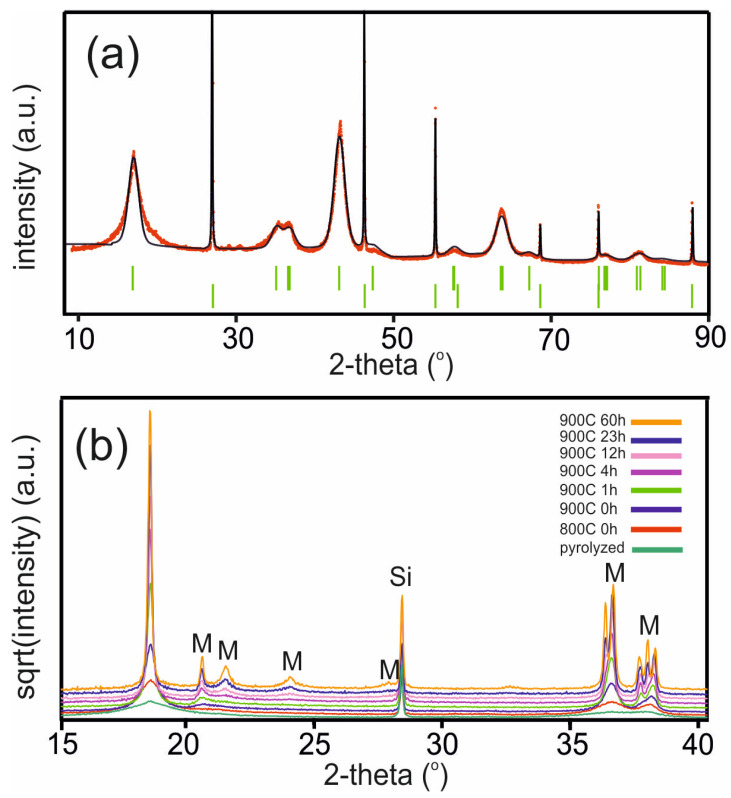
(**a**) The powder pattern of pyrolyzed, but not calcined, Ni35-Al01. The black line corresponds to a fit of an R phase. The sharp peaks and lower reflection markers are for the Si internal standard. (**b**) The powder patterns for samples at different holding times t and temperatures. The positions of isolated M phase reflections and Si are indicated.

They were performed on pyrolyzed, but not calcined, powders in order to replicate calcination conditions. The materials were held at 900 °C for t = 0, 1, 4, 23, and 60 h. The pyrolyzed starting material, not calcined, shows a powder pattern, see Figure 4a, which can be roughly modeled by an R phase. The peaks are broad and correspond to a size broadening of ~50 Å large domains. There are only a few unidentified weak additional peaks, showing that the powder has essentially a homogeneous distribution of elements.

**Figure 5:**
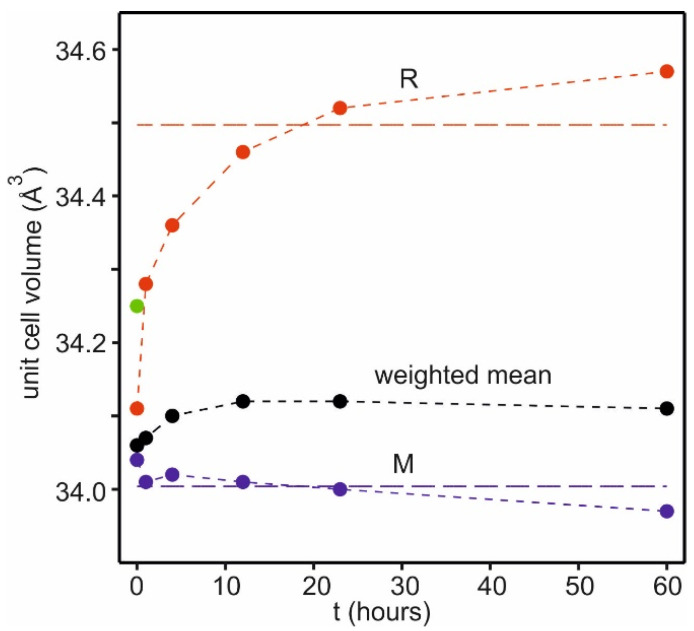
Normalized unit cell volumes V of the M (blue) and R (red) phases for different holding times t at 900 °C for Ni35-Al01. The mean volume (black) is calculated using a constant value of 76 mol% M derived from NPD data [2]. The dashed lines correspond to the unit cell parameters found previously for Ni35-Al01 for a calcination time of 6 h [2]. The unit cell volume for the pyrolyzed starting material is shown by the filled green circle.

The powder patterns for the samples at different holding times t and temperatures are shown in Figure 4b. The pattern of a sample heated to 800 °C is basically similar to that of the starting pyrolyzed material shown in Figure 4a, but with slightly sharper peaks. It is difficult to model, as it is of a powder at the transition from R to a mixture of R + M. Already at 900 °C, with no holding time, t = 0, the pattern is that of a mixture of M and R. As the holding time at 900 °C increases, the peak widths decrease and the normalized unit cell volumes for M and R are found to diverge, as shown in Figure 5 (cf. Tables S2 and S3).

As the unit cell volumes for M and R diverge, V for R increases, and that for M remains more constant. The mean cell volume shows only a small increase for t ≤ 4 h. The estimated fraction of M remains roughly constant, ~75 wt% (cf. Table S4). The results suggest a progressive enrichment of the larger Ni^2+^ in a constant fraction of the R phase via the substitution mechanism 3Ni^2+^ ↔ 2Li^+^ + 1Mn^4+^.

**Figure 6:**
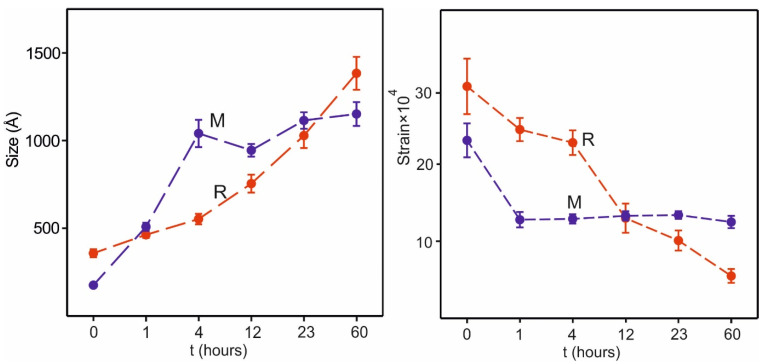
X-ray domain size (**left**) in Å and strain (**right**) for M (blue) and R (red) at different heat treatment times t.

The derived X-ray domain size and strain (cf. Table S5) are shown in Figure 6. The size increases with t for both M and R. The strain shows a constant strain for M after the first hour and a decreasing strain for R.

### 3.2. Heat Treatments of Al-Doped Ni35 at 900 °C for Long Times

**Figure 7:**
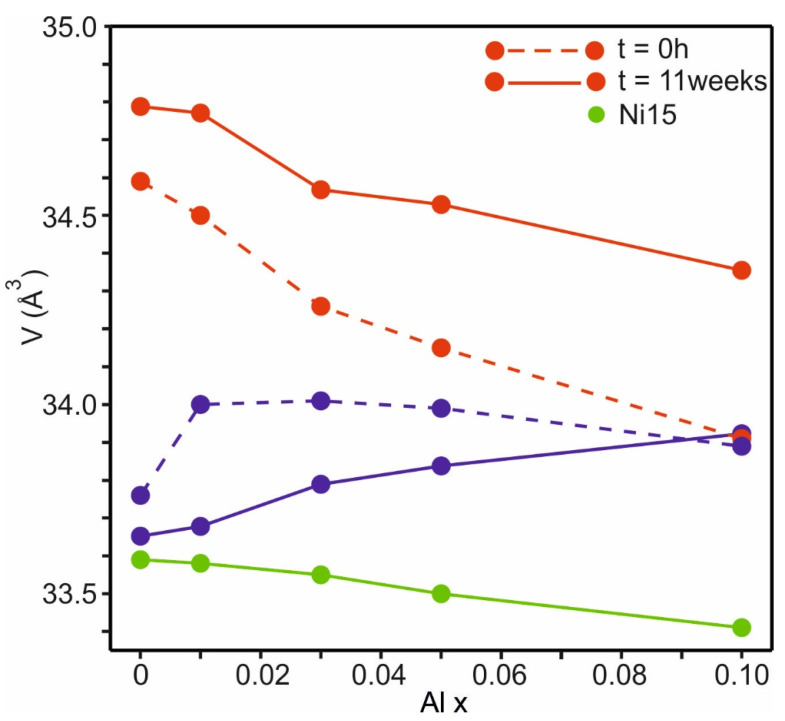
Normalized unit cell volumes for Al-doped Ni35 samples; M = blue lines; R = red lines; dashed lines = reference [2]; solid lines = after 11 weeks at 900 °C. Unit cell volumes for Al-doped Ni15 samples are shown by filled green circles.

These were performed on pressed pellets, and not on powders as the short heat treatments, of previously characterized materials [2], i.e., already calcined for 6 h at 900 °C. The samples were held at 900 °C for t = 14 h, 43 h, 1 week, 3 weeks, and 11 weeks. The evolution of V for M and R with heat treatment time is described for each x in the Supplementary Materials (cf. Figure S6 and Tables S6 and S7). The estimated fractions of Ni in M and the mole fractions of M are given in Tables S8 and S9. The normalized unit cell volumes for the untreated Ni35 samples, t = 0, calcinated after pyrolysis for 6 h [2], and the Ni35 samples held at 900 °C for t = 11 weeks are shown in Figure 7, together with the unit cell volumes for the Al-doped Ni15 samples (cf. Table S11).

The unit cell volume increases with the increasing incorporation of Ni^2+^ in the phases and is roughly a direct measure of their Ni contents. M contains less Ni, and thus more Mn, than R [2,11,12]. The marked increase in V for the untreated Ni35 between Al00 and Al01 is, thus, due to the fact that the addition of Al effects a higher Ni content in M. For the untreated samples, V for M and R converge with increasing Al content, indicating an equalization of Ni content, and the two phases are not distinguishable for Al10 [2]. After 11 weeks at 900 °C, V for M decreases, in particular for lower x values, due to a decrease in Ni content via the substitution 3Ni^2+^ ↔ 2Li^+^ + 1Mn^4+^, as also verified by Rietveld refinements, Table S8. Correspondingly, there has to be an increase in Ni for R, although this cannot be quantified by refinements using XRPD data, as Mn and Ni are about equally strong X-ray scatterers. The heat treatment, thus, effects a transference of Ni from M to R and a corresponding divergence of unit cell volumes.

For x ≤ 0.03, the estimated mean V shows a smaller increase with heat treatment time t. The increase is largest for Al10, ~0.26 Å^3^. A similar increase is observed upon heat-treating Ni15-Al01 M. The reason is not known, and we refrain from speculation. ^7^Li solid-state magic-angle spinning nuclear magnetic resonance (MAS NMR) spectroscopy measurements, described in Supplementary Materials Section S3.2.2 (cf. Figure S7), did not provide further enlightenment. The two signals from Li in the Li layers and Li in the transition metal layers are discernible with the corresponding isotropic chemical shifts of 710 and 1420 ppm, respectively. However, only minute changes for different heat treatment times t were observed. For the undoped sample after 11 weeks of heat treatment, signals appeared to be narrower, which could be connected to changes in grain morphology and smaller signal broadening due to bulk magnetic susceptibility (BMS) effects. For the Al10 11-week counterpart, signal intensity from diamagnetic Li (0 ppm), e.g., as from Li_2_CO_3_, increased; however, this effect is minor in relation to the total NMR signal integral of the sample. Note that individual contributions from the M and R phases are not resolved in ^7^Li MAS NMR, and therefore, small changes in cation composition and/or oxidation state variations needed to give quantifiable changes in V could be hidden in such broad ^7^Li NMR patterns.

For Ni35, the compositions of the M phases were determined by refining the amount of incorporated Ni by the mechanism 3Ni^2+^ ↔ 2Li^+^ + Mn^4+^ in Li(Li_1/3_Mn_2/3_)O_2_ [2]. The thus determined M composition for Al00 Li_1.25_Mn_0.62_Ni_0.13_O_2_ is very similar to the nominal Ni15 composition Li_1.26_Mn_0.61_Ni_0.15_O_2_. The unit cell volumes for these samples are in accordance also very similar.

**Figure 8:**
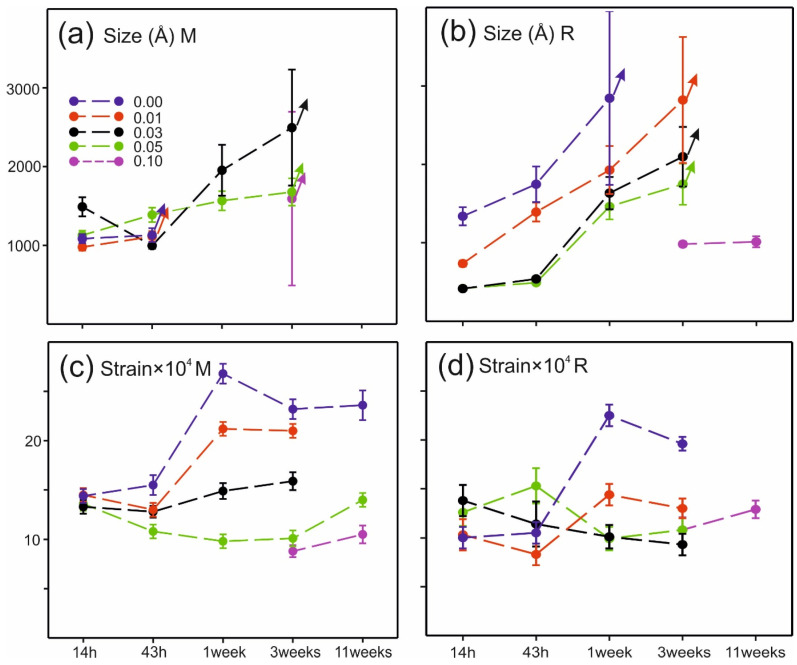
X-ray domain size for M (**a**) and R (**b**) and strain for M (**c**) and R (**d**). No points are shown (i) for domain size when the peak width is instrumental resolution-limited, (ii) for domain size or strain where the M and R phases are not distinguishable in the powder patterns (Al10 for t ≤ 1 week), and (iii) for samples with anisotropic size broadening for R (Al00, Al01, and Al03 for t = 11 weeks). The arrows show points for which no size broadening is found upon subsequent heat treatments.

The sample broadening in the powder patterns is dominated by strain broadening (cf. S3.2.3). The first, (001)_M_/(003)_R_, peak shows only a small sample broadening, while there is a considerable increasing peak broadening with increasing 2θ. The derived X-ray domain sizes and strains (cf. Table S10) are shown in Figure 8.

The X-ray domain size increases for both M and R with heat treatment time t. The domain sizes are smaller for R than for M. The increase in size decreases clearly with Al content, implying that Al doping slows down kinetics.

The strain decreases clearly for M with increasing Al content, while no clear trends are seen for R.

For x ≤ 0.03 and t = 11 weeks, peaks from R showed an anisotropic Lorentzian sample broadening with sharper (00*l*) peaks. A similar anisotropic peak broadening has been observed for Al-substituted lithium nickel oxides LiNi_1−y_Al_y_O_2_, attributed to a segregation tendency of Al and Ni [15].

### 3.3. Heat Treatments of Ni15-Al01

The powder patterns for the as-received sample and pelletized samples held at 900 °C for 1 week and 1000 °C for 24 h were found to be practically identical. The unit cell volumes (cf. Table S11) were, however, found to have increased slightly, by ~0.10 Å^3^, as found also for the Ni35 samples. The heat treatments show that the M phase is stable upon further holding at 900 °C and at 1000 °C, and suggests also that there is no significant loss of Li.

A sample held at 1200 °C for 24 h was found to contain ~64 wt% of M, ~35 wt% of a cubic rock-salt phase RS, and ~1 wt% of orthorhombic LiMnO_2_ (ICSD entry 95640). The unit cell parameter of the spinel phase is similar to that reported for the composition Li_0.58_Mn_1.02_Ni_0.40_O_2_ [11]. The decomposition of the original M phase is, thus, accompanied by a reduction of some of the Mn^4+^ to Mn^3+^, as evidenced by the forming of LiMnO_2_.

The pattern of a sample held at 1100 °C for 24 h showed the same peaks as the sample held at 1000 °C, but with broader peaks. We interpret this as evidence for an onset of a decomposition of the kind seen for the sample heated to 1200 °C.

### 3.4. Refinements of Ni15-Al00/Al05/Al10 Structures from NPD Data

The XRPD and NPD patterns showed peaks attributable to a single M phase and minor amounts of Li_2_CO_3_.

The unit cell volume decreases linearly with an increasing Al content. The ionic radii for Mn^4+^ and Al^3+^ are very similar, 0.53 and 0.535 Å, respectively [16], but a small decrease in unit cell volume may occur by small changes in compositions taking place to realize cation–anion and charge balance.

**Figure 9:**
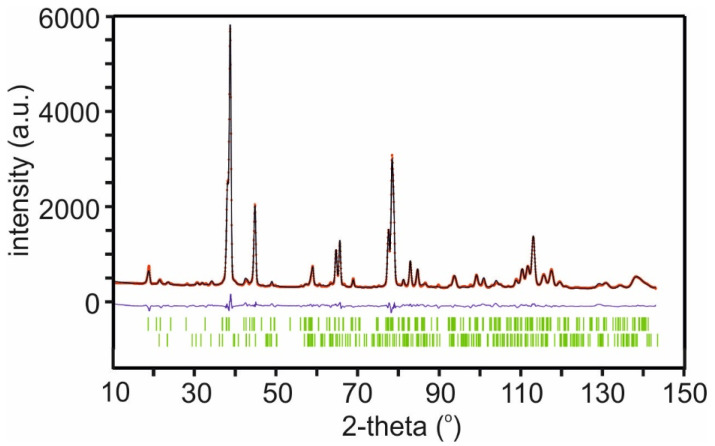
X Observed (red), calculated (black), and difference (blue) NPD patterns for the Rietveld refinement for Ni15-Al10 from NPD data. The lower reflection markers are for Li_2_CO_3_.

For Ni35, the incorporation of Ni in M was refined, but this is not possible for the single-phase Ni15 samples if the nominal compositions are to be kept. Instead, Mn, Ni, Al, and Li were placed on the smaller 4g site; the remaining Ni and Li on the 2b site, with the occupations complying with the nominal compositions; and a transference of Li and Ni between the 4g and 2b sites then refined. The Li layers were kept unaffected. The best fits, with R_F_ values 4–4.5%, were obtained by having the 4g sites occupied by Mn^4+^, Al^3+^, and Ni^2+^, and the 2b sites occupied by the remaining Ni^2+^ and Li^+^. Refinements with a slightly decreased the total Li content produced small improvements in fits. However, these improvements were very marginal, and not quantitatively reliable. Further data are given in Supplementary Materials Section S4. The Rietveld fit for Ni15-Al10 is shown in Figure 9.

## 4. Discussion

The results of the heat treatment experiments show that for these Ni35 nano-powder samples of the mixtures of M and R, phase compositions and fractions change with time upon heat treatment at 900 °C in air, and equilibration takes a long time. There is also a pronounced grain growth, implying a migration of cations between phases and grains. We see no indications of Li loss upon heat treatment at 900 °C, e.g., the formation of other phases, in addition to the Li_2_O losses that occur already after pyrolysis and calcination. For the longer heat treatments of the Ni35 samples, there is a small increase in the estimated mean unit cell volume with time, which also increases with the Al content. The Ni15-Al01 sample also shows an increase in unit cell volume upon heat treatment. Only very small changes in composition and/or oxidation states are needed to affect the unit cell volume (cf. Supplementary Materials Section S5.1).

The unit cell volumes for M and R for Ni35 diverge upon heat treatment, implying a progressive decrease in Ni in M and the enrichment of Ni in R. For Al10, M and R are not distinguishable by XRPD for t ≤ 1 week, but segregate to a two-phase mixture of M + R for longer heat treatments.

It is notable that the apparent X-ray domain sizes are large: ~1000 Å after heat treatment for only 4 h and growing upon further heat treatments. They are of comparable dimensions as the grain sizes, even for compositions for which considerably smaller phase regions are found by analytical STEM [1,2,3].

Nano-domains have been found to exist in the Li-rich cathode materials of the present type by several techniques: analytical electron microscopy and EXAFS for Li_1.2_Co_0.4_Mn_0.4_O_2_ [17], NMR for Li_1.2_Mn_0.61_Ni_0.18_Mg_0.01_O_2_ [18], resonant X-ray diffraction spectroscopy and X-ray absorption spectroscopy for Li_1.2_Ni_0.2_Mn_0.6_O_2_ [19], and analytical STEM for the present Ni35 samples [2]. In all these studies long-range order is preserved, i.e., the nano-domains do not produce sample size broadening in XRPD patterns. We believe that this is connected with the observation that the phases have a common oxygen lattice in much larger domains, frequently extending over whole grains.

In the previous extensive study of the Ni15 cathode materials [3], the grains were concluded to exhibit a core–shell structure of two phases, denoted tentatively as M and R, with M at the center, while the powder diffraction data could be accounted for by only one M phase. The structures and compositions of M and R remained, however, largely unclear, and their existence and distribution were concluded from the analyses of NMR and HyDF/EELS data, both local probes. It seems possible that the core–shell structure is one of an unequal distribution of the nano-domains of M with different local orderings.

## 5. Conclusions

The short-term heat treatments of the powders of Ni35-Al01 at 900 °C show how the M and R phases evolve within the ~0.1 μm sized grains of the pyrolyzed materials upon varying the calcination time. With increasing holding time, the unit cell volumes of M and R diverge as the unit cell volume for R increases due to an increasing Ni content, accomplished by the substitution mechanism 3Ni^2+^ ↔ 2Li^+^ + 1Mn^4+^. The unit cell volume for the M phase decreases, but less as it is the dominating phase. Concomitantly, the X-ray domain size increases for both M and R. The strain decreases for R, but remains more constant for M.

The longer-term heat treatments on the pressed pellets of Ni35 with different Al contents show how the phase assembly progresses towards equilibrium. There is a pronounced grain growth, with a grain size of ~1 μm attained upon heat treatment at 900 °C for 3 weeks. The unit cell volumes of M and R diverge due to an enrichment of Ni in R, as for the short heat treatments above. At the same time, the X-ray domain sizes increase further and are larger for M than for R. The domain size decreases with increasing Al content. This indicates that the doping by Al slows down kinetics and the rate towards equilibration, which could be of importance for use as a cathode material.

It is noteworthy that the X-ray domain size is much larger than the phase domains observed by electron microscopy.

The structure refinements of the Ni15 samples verify that the structures for these mono-phasic M phases are very similar to those derived for the corresponding M phases found in the mixtures of M and R in the Ni35 samples. This, in turn, validates the proposed mechanism whereby Ni is incorporated into the M structure; one Ni substitutes for one Mn on the 4g site and two Ni substitutes for two Li on the 2b site.

Overall, the results show the inherent difficulties in producing reproducible samples of these types of cathode materials and thereby the identification of factors important for the electro-chemical performances.

## Data Availability

The original contributions presented in the study are included in the article and Supplementary Materials; further inquiries can be directed to the corresponding author.

## Supplementary Materials

The following supporting information can be downloaded at: https://www.mdpi.com/article/10.3390/ma17246056/s1, Figure S1: Octahedral layer stackings in the ideal Li_2_MnO_3_ M structure and the ideal LiMn_1/2_Ni_1/2_O_2_ R structure; Figure S2: Secondary electron images of cross section polished surfaces of Ni35-Al05 and Ni35-Al10 heat treated for 3 weeks; Figure S3: As recorded XRPD pattern for Ni35-Al01 heat treated for 3 weeks; Figure S4: Rietveld plot for Ni35- Al01 heat treated for 3 weeks; Figure S5: Rietveld plots for Ni35-Al01 heat treated at 900 °C for 0 h and 60 h; Figure S6: Normalised unit cell volumes as a function of heat-treatment time t for Ni35 with different Al contents; Figure S7: ^7^Li MAS NMR spectra of undoped (left panel) and 10% Al doped specimens collected for as synthesized and heat treated samples for 11 weeks; Figure S8: Calculated actual and with no sample broadening powder patterns (15° ≤ 2θ ≤ 40) for Ni35-Al01 heat treated for 3 weeks; Figure S9: Calculated actual and with no sample broadening powder patterns (75° ≤ 2θ ≤ 100°) for Ni35-Al01 heat treated for 3 weeks; Figure S10: Peak half-widths as a function of 2θ for M and R for Ni35-Al01 heat treated for 3 weeks; Figure S11: Unit cell volume V vs. x for Ni15 M phases from PXRD; Figure S12: Unit cell volume V vs. the mean Shannon-Prewitt of cations in M and R phase. Table S1: Crystallographic atom sites in the ideal M structure; Table S2: Unit cell parameters for M for Ni35-Al01 heat treated at 900 °C; Table S3: Unit cell parameters for R. for Ni35-Al01 heat treated at 900 °C; Table S4: χ2, structure R-factors and estimated fraction of M for Ni35-Al01 heat treated at 900 °C; Table S5 Size and strain for short temperings of Ni35-Al01 heat treated at 900 °C; Table S6: Unit cell parameters for M and different x; Table S7: Unit cell parameters for R and different x; Table S8: Estimated fraction in % of Ni relative to (Mn + Ni) from XRPD data in M for Al doped Ni35; Table S9: Estimated mole fraction of M in % for Al doped Ni35 from XRPD data; Table S10: Size and strain for M and R for long term heat treatments of Ni35-Al; Table S11: Unit cell parameters for Ni15 M phases from PXRD data; Table S12: Strain and size for Ni15 M phases; Table S13: Compositions of 4g and 2b sites for refinement sets A, B and C; Table S14: Reliability indices for refinements for Ni15 M phases using NPD data; Table S15: Unit cell parameters for Ni15 M phases from NPD data; Table S16: Atomic coordinates for Al doped Ni15 M phases from NPD data; Table S17: Metal-O distances in the transition metal layer for Ni15 M phases from NPD data; Table S18: Li-O distances in the Li metal layer for Al doped M phases; Table S19: Bond valence sums (BVS) for O atoms.
