# Supplementary material for: Phase Evolution of Li-Rich Layered Li-Mn-Ni-(Al)-O Cathode Materials upon Heat Treatments in Air
Source: Materials (Basel). 2024 Dec 11;17(24):6056. doi: 10.3390/ma17246056 (PMC11728093; doi:10.3390/ma17246056)
Supplement: Supplementary file 1 [file materials-17-06056-s001.zip › materials-3326046-supplementary.pdf]

Supplementary information for

Phase Evolution of Li-Rich Layered Li-Mn-Ni-(Al)-O Cathode Materials upon Heat Treatments in Air

Jekabs Grins, Aleksander Jaworski, Leif Olav Jøsang, Jordi Jacas Biendicho and Gunnar Svensson

S1. Introduction

The octahedral layer stackings of the ideal M and R structures are illustrated below.

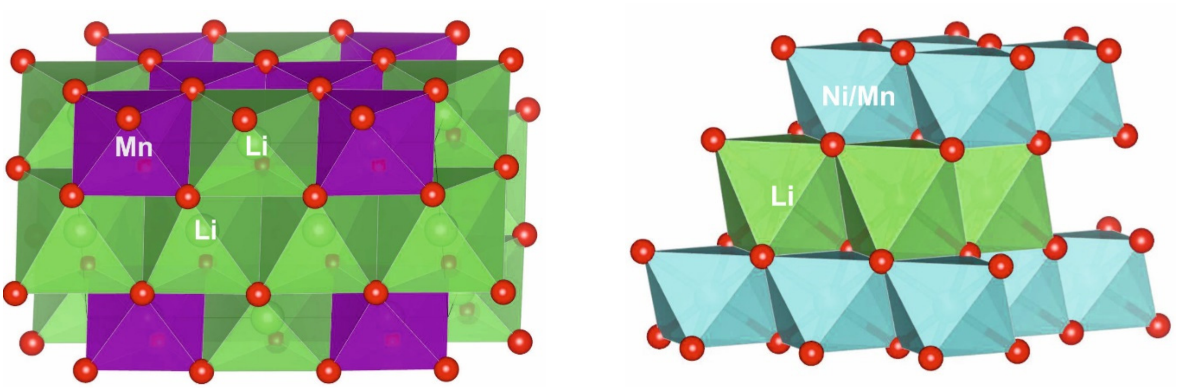

Figure S1. Octahedral layer stackings in (left) the ideal Li_2MnO_3 M structure and (right) the ideal $LiMn_{1/2}Ni_{1/2}O_2$ R structure.

In the ideal M structure, the transition metal, TM, layers contain Mn on the 4g site and Li on the 2b site. Ni may be incorporated in the structure on these sites. The Li layer has only Li, on the 4h and 2c sites. The structure has 7 refinable atomic positions.

Table S1. Crystallographic atom sites in the ideal M structure.

Wyckoff	Element	*x*	*y*	*z*
2b	Li	0	½	0
2c	Li	0	0	½
4g	Mn	0	0.17	0
4h	Li	0	0.66	½
4i	O	0.22	0	0.22
8j	O	0.25	0.33	0.22

In the ideal R structure, Mn and Ni reside on the 3b site (0, 0, ½) and Li on the 3a site (0, 0, 0). The structure has one refinable z-coordinate for O at the 6c site (0, 0, z = ~0.25). The structure allows for a flexible composition and the transition metal layer may contain Li and the Li layer may contain Ni.

S2. Materials and Methods

S2.1. Cross section polished surfaces of Ni35-Al05 and Ni35-Al10.

No compositional variations could be observed by SEM for Ni35-Al05 and Ni35-Al10 heat treated for 3 weeks, neither by EDS or BSE contrast.

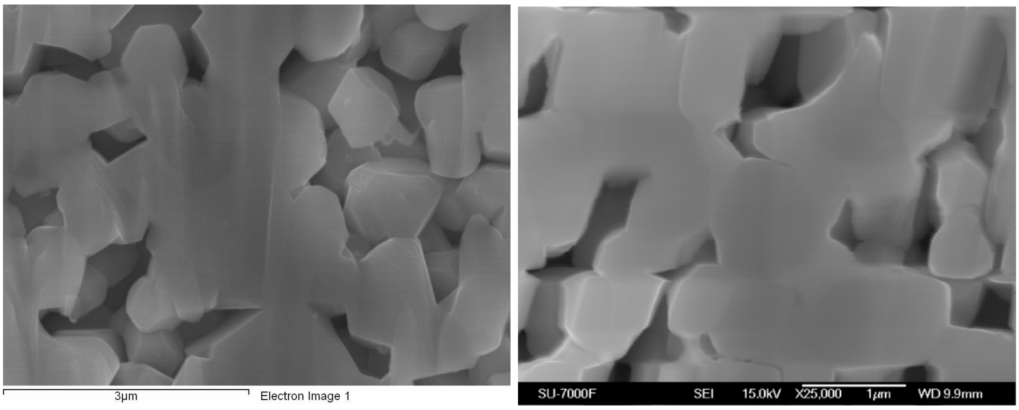

Figure S2. Secondary electron images of cross section polished surfaces of Ni35-Al05 (left) and Ni35-Al10 (right) heat treated for 3 weeks.

S2.2. Collection of XRPD data

XRPD patterns were collected using a Panalytical PRO MPD diffractometer (CuKα radiation). The powder samples were spread thinly on Si zero background discs. NIST 640c Si was added as an internal standard in order to correct the 2θ-scale. Data were collected in the 2theta-range 15 to 130° with a step length of 0.016° and a total recording time 4 h. Maximum peak intensities were about 50000 counts. An example of a raw powder pattern is shown below. For refinements, the data were converted to fixed slit data and the Kα2 component removed. The 2theta-scale was corrected using Panalyticals HighScore+ evaluation software and a second order polynom. The zero-point and Si unit cell parameter were in addition then refined during the Rietveld refinements and the unit cell parameters obtained from the refinements were finally multiplied by the correct unit cell parameter for Si, taken here as 5.43088 Å, divided by the refined parameter. Alpha-2 peaks were for the refinements stripped using HighScore+.

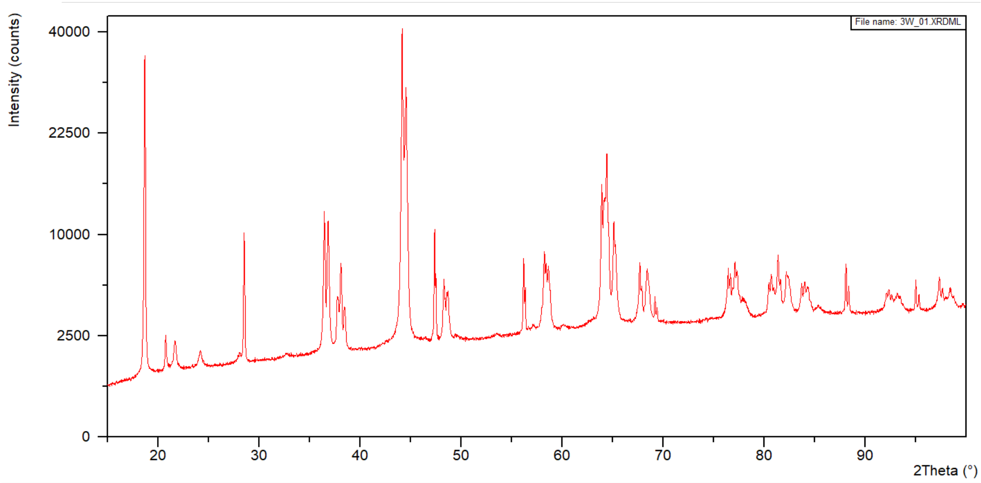

Figure S3. As recorded XRPD pattern for Ni35-Al01 heat treated for 3 weeks.

S2.3. Structure refinements

In the refinements, the NPROF = 5 pseudo-Voigt profile shape function was used, which has 4 refinable parameters; U, V, W and eta. e.s.d.'s were obtained by multiplying the corresponding values from the refinement by a factor accounting for serial correlation in the data, as suggested by Berar and Lelann, being for the present samples ~ 5-6, and $\sqrt{2}$ (for the error for the Si unit cell parameter).

About 25-30 parameters were refined, including atom position parameters for M and R. The main objective for the refinements using XRPD data was, however, not to extract information on structures but to obtain accurate unit cell parameters. Estimated phase fractions are approximate. An example of a Rietveld fit is shown below.

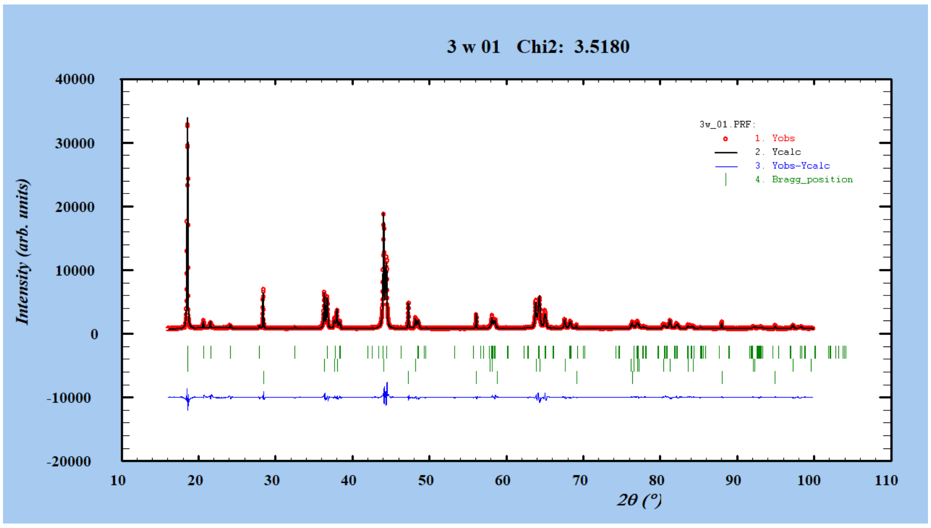

Figure S4. Rietveld plot for Ni35- Al01 heat treated for 3 weeks.

S2.4. Size and strain analysis using FullProf

The basis for size/strain analysis using the Rietveld method in FullProf is the Thomson-Cox-Hastings (TCH) pseudo-Voigt (Pv) function, which describes the peak profiles as sums of a Lorentzian (L) peak and a Gaussian (G) peak, according to Pv(x) = η·L(x) + (1-η)·G(x). The Lorentzian and Gaussian peak components are for the basic expression (and for isotropic broadening) defined as:

$$H_G^2 = U\cdot\tan^2(\theta) + V\cdot\tan(\theta) + W + Ig/\cos^2(\theta)$$

$$H_L = X\cdot\tan(\theta) + Y/\cos(\theta)$$

From H_G and H_L the total peak width H and the mixing parameter η is calculated from

$$H = (H_G^5 + 2.69269H_G^4H_L + 2.42843H_G^3H_L^2 + 4.47163H_G^2H_L^3 + 0.07842H_GH_L^4 + H_L^5)^{0.2}$$

$$\eta = 1.36603(H_L/H) - 0.47719(H_L/H)^2 + 0.11116(H_L/H)^3$$

Instead of using H_L and H_G in the broadening analysis one uses the corresponding integral breadths β_L and β_G (widths of rectangles with the same area).

$$\beta_G = \pi\cdot H_G/2$$

$$\beta_L = (H_L/2)\cdot\sqrt{(\pi/\ln(2))}$$

The volume-weighted crystallite size $\langle D\rangle_v$ and strain ε is then usually calculated by

$$\langle D\rangle_v = \lambda/\beta_L\cdot\cos(\theta)$$

$$\varepsilon = (1/4)\cdot\beta_G/\tan(\theta)$$

The fundamental reasoning is that size broadening should roughly effect all reflection equally (and only show a small 1/cos(θ) dependence), while a strain broadening should increase proportionally with tan(θ). The integral peak breadths β_L and β_G must first be corrected for the broadening from the instrument itself by $\beta_L = \beta_{Lobs} - \beta_{Lstd}$ and $\beta_G^2 = \beta_{Gobs}^2 - \beta_{Gstd}^2$. The instrumental broadening was here estimated from the peak widths of the NIST 640c Si standard. After specifying the instrumental broadening, four parameters can be refined: U, X, Y, and Ig. The strain is $\propto U^{1/2}$ and X, and size $\propto$ Y and $Ig^{1/2}$. When a refined value turned (slightly) negative, it was set to zero. Refinements without the instrumental broadening specified was taken as starting sets. In addition to the sample broadening parameters, scale factors and overall temperature factors were refined.

S2.5. Errors in domain size and strain

Following Bakhar *et al.*, *Acta Cryst. B* **73** (2017) 1095-1104, the Gaussian and Lorentzian contributions to the strain and size parameters can be expressed in terms of the refinable parameters U, X, Y, Ig as;

$$\varepsilon_G = \frac{1}{4}\left(\frac{\pi}{\ln 2}\right)^{1/2}\frac{\pi}{360}\cdot U^{1/2} \qquad \sigma_{\varepsilon_G} = \frac{1}{4}\left(\frac{\pi}{\ln 2}\right)^{1/2}\frac{\pi}{360}\cdot\frac{1}{2}U^{-1/2}\cdot\sigma_U$$

$$\varepsilon_L = \frac{1}{4}\frac{\pi^2}{360} \cdot X \qquad \sigma_{\varepsilon_L} = \frac{1}{4}\frac{\pi^2}{360} \cdot \sigma_X$$

$$D_{VG} = \frac{1}{4}(\pi \ln 2)^{1/2}\left(\frac{360\,\lambda}{\pi^2}\right) \cdot \left(\frac{1}{I_G}\right)^{1/2} \qquad \sigma_{D_{VG}} = \frac{1}{4}(\pi \ln 2)^{1/2}\left(\frac{360\,\lambda}{\pi^2}\right) \cdot \frac{1}{2}\left(\frac{1}{I_G}\right)^{3/2} \cdot \sigma_{I_G}$$

$$D_{VL} = \left(\frac{360\,\lambda}{\pi^2}\right) \cdot \left(\frac{1}{Y}\right) \qquad \sigma_{D_{VL}} = \left(\frac{360\,\lambda}{\pi^2}\right) \cdot \left(\frac{1}{Y}\right)^2 \cdot \sigma_Y$$

When η is either 1 or 0, *i.e.*, when there is either only a Gaussian or only a Lorentzian contribution, the errors in strain or size can be calculated rather easily from the errors in the refined parameters. The errors are proportional to;

$$\sigma_{\varepsilon_G} \propto \left(\frac{1}{\varepsilon_G}\right)\sigma_U \qquad \sigma_{\varepsilon_L} \propto \sigma_X \qquad \sigma_{D_{VG}} \propto D_{VG}^3 \cdot \sigma_U \qquad \sigma_{D_{VL}} \propto D_{VG}^2 \cdot \sigma_{I_G}$$

The principal components “should” be from U (strain) and Ig (size).

S3. Results

S3.1. Short term heat treatments at 900 °C of sample Ni35-Al01.

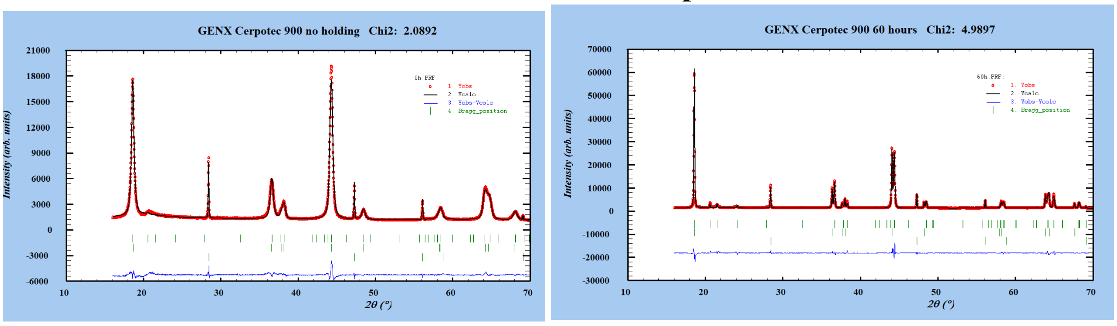

Figure S5. Rietveld plots for Ni35-Al01 heat treated at 900°C for (left) 0 h and (right) 60h.

Table S2. Unit cell parameters for M for Ni35-Al01 heat treated at 900 °C. Statistical errors are ~0.003 Å, ~0.005 Å, ~0.003 Å and ~0.02° for *a*, *b*, *c* and β.

Time(h)	*a*	*b*	*c*	*β*	V*
0	4.969	8.638	5.039	109.16	34.04
1	4.976	8.613	5.042	109.20	34.01
4	4.978	8.613	5.042	109.22	34.02
12	4.972	8.621	5.041	109.18	34.01
23	4.972	8.618	5.039	109.15	34.00
60	4.967	8.608	5.038	109.07	33.93

* Normalised.

Table S3. Unit cell parameters for R. for Ni35-Al01 heat treated at 900°C. Statistical errors are ~0.0006 Å and ~0.006 Å for *a* and *c*.

Time(h)	*a*	*c*	V*
pyrolized	2.889	14.212	34.25
0	2.881	14.232	34.11
1	2.885	14.270	34.28
4	2.867	14.283	34.36
12	2.890	14.296	34.46
23	2.891	14.303	34.52
60	2.892	14.312	34.57

* Normalised.

Table S4. $\chi 2$, structure R-factors and estimated fraction of M for Ni35-Al01 heat treated at 900 °C.

Time(h)	$\chi 2$	$R_F(M)$	$R_F(R)$	wt% M*
0	2.0	5.3	2.4	75
1	1.7	5.7	1.7	59
4	2.4	5.4	2.6	69
12	3.0	6.6	2.9	74
23	3.2	7.2	3.2	72
60	5.0	8.7	2.9	63

* NPD [2] gives 76 mol% M for a calcination time of 6h.

Table S5. Size and strain for short temperings of Ni35- Al01 heat treated at 900 °C.

sample	Size (Å)	Error	Strain·10^4	Error
Start	52	0.5		
800 M	82	0.2	63	5.9
0h M	176	6	23	2.2
1h M	509	23	13	1.0
4h M	1041	78	13	0.6
12h M	945	36	13	0.6
23h M	1109	47	13	0.5
800 R	248	22	43	6.0
0h R	356	23	30	3.6
1h R	463	18	25	1.5
4h R	553	30	23	1.6
12h R	755	51	14	1.9
23h R	1036	71	10	1.3
60 h R	1384	94	6	0.9

S3.2. Long term heat treatments of Ni35 – Alx at 900 °C.

S3.2.1. Unit cell parameters, estimated fractions of Ni in M and estimated mole fractions of M

The evolution of unit cell volumes with tempering time for Ni35 and different x is shown below. There is a small increase in the estimated mean unit cell volume with time, which also seems to increase with Al content.

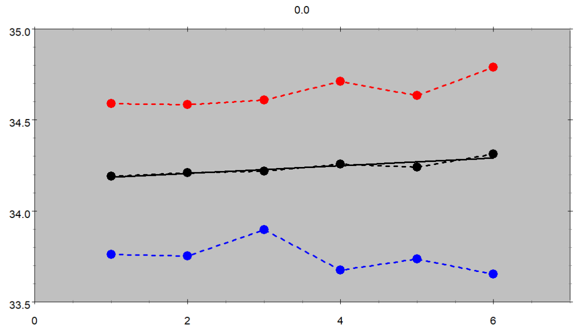

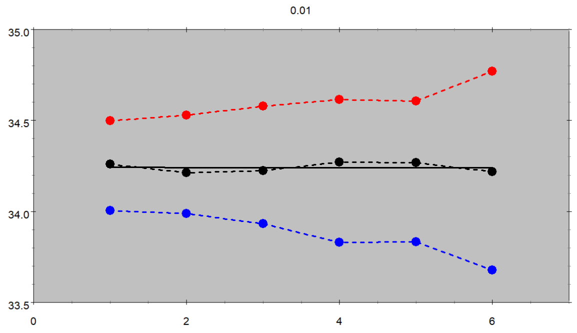

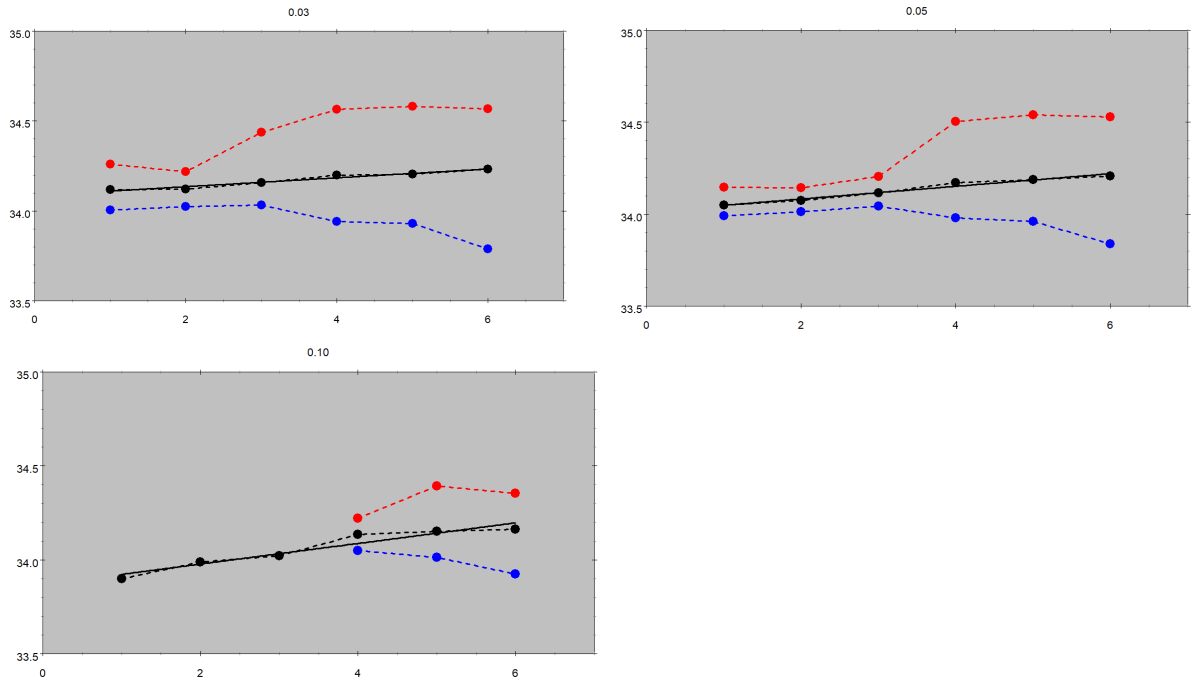

Figure S6. Normalised unit cell volumes as a function of heat-treatment time t for Ni35 with different Al contents x; (red) = for R, (blue) = for M, (black) = estimated average. The integral x-axis numbers correspond to consecutive tempering times of 0, 14 h, 43 h, 1 week, 3 weeks, and 11 weeks.

Table S6. Unit cell parameters for M and different x. Statistical errors are ~0.003 Å, ~0.005 Å, ~0.003 Å and ~0.02° for *a*, *b*, *c* and β.

x Al	Sample	*a* (Å)	*b* (Å)	*c* (Å)	*β* (°)	V* (Å^3)
0.0						
	ref [2]	4.979	8.565	5.034	109.19	33.76
	14h	4.968	8.611	5.038	109.09	33.95
	43h	4.965	8.604	5.038	109.07	33.90
	1week	4.950	8.573	5.045	109.29	33.68
	3weeks	4.954	8.583	5.045	109.32	33.74
	11weeks	4.949	8.563	5.049	109.32	33.65
0.01						
	ref [2]	4.976	8.616	5.040	109.22	34.00
	14h	4.973	8.616	5.039	109.15	33.99
	43h	4.968	8.609	5.037	109.08	33.93
	1week	4.961	8.594	5.037	109.06	33.83
	3weeks	4.960	8.594	5.038	109.05	33.83
	11weeks	4.950	8.567	5.050	109.34	33.68
0.03						
	ref [2]	4.976	8.617	5.040	109.22	34.01
	14h	4.974	8.621	5.041	109.15	34.02
	43h	4.976	8.620	5.039	109.13	34.03
	1week	4.968	8.607	5.039	109.05	33.94
	3weeks	4.967	8.606	5.039	109.04	33.93
	11weeks	4.955	8.586	5.040	109.00	33.79
0.05						
	ref [2]	4.971	8.616	5.038	109.24	33.99
	14h	4.980	8.609	5.042	109.22	34.14
	43h	4.979	8.616	5.045	109.28	34.04
	1week	4.973	8.608	5.048	109.35	33.98
	3weeks	4.972	8.605	5.048	109.36	34.54
	11weeks	4.963	8.586	5.050	109.35	33.84
0.10						
	ref [2]**	*4.958*	*8.666*	*5.016*	*109.32*	*33.89*
	14h**	*4.974*	*8.620*	*5.037*	*109.14*	*34.00*

	43h**	*4.976*	*8.622*	*5.038*	*109.12*	*34.04*
	1week	4.984	8.649	5.044	109.21	34.22
	3weeks	4.975	8.611	5.050	109.35	34.01
	11weeks	4.967	8.596	5.052	109.35	33.92

* Normalised.
**Unreliable.

Table S7. Unit cell parameters for R and different x. Statistical errors are ~0.0006 Å and ~0.006 Å for *a* and *c*.

x Al	Sample	*a* (Å)	*c* (Å)	V* ($Å^3$)
0.0				
	Ref [2]	2.896	14.287	34.59
	14h	2.893	14.311	34.58
	43h	2.894	14.316	34.61
	1week	2.897	14.329	34.71
	3weeks	2.894	14.325	34.63
	11weeks	2.899	14.339	34.79
0.01				
	Ref [2]	2.893	14.282	34.50
	14h	2.892	14.303	34.53
	43h	2.893	14.313	34.58
	1week	2.894	14.321	34.62
	3weeks	2.893	14.322	34.61
	11weeks	2.898	14.340	34.77
0.03				
	Ref [2]	2.884	14.271	34.26
	14h	2.881	14.284	34.22
	43h	2.889	14.296	34.44
	1week	2.892	14.315	34.57
	3weeks	2.893	14.318	34.58
	11weeks	2.891	14.331	34.57
0.05				
	Ref [2]	2.879	14.267	34.15
	14h	2.878	14.280	34.14
	43h	2.880	14.287	34.21
	1week	2.890	14.312	34.51
	3weeks	2.891	14.316	34.54
	11weeks	2.889	14.329	34.53
0.10				
	Ref [2]**	*2.8694(2)*	*14.2663(20)*	*33.908*
	14h**	*2.8716*	*14.2732*	*33.976*
	43h**	*2.8725*	*14.2807*	*34.016*
	1week	2.8731	14.2893	34.050
	3weeks	2.8854	24.3101	34.393
	11weeks	2.8827	14.3212	34.355

* Normalised.
**Unreliable.

Table S8. Estimated fraction in % of Ni relative to (Mn + Ni) from XRPD data in M for Al doped Ni35.

	Al00	Al01	Al03	Al05	Al10
Ref [2]*	18	27	28	26	-
14h	32	34	37	38	-
43h	30	35	36	39	-
1week	23	28	33	35	-
3weeks	26	28	33	34	39
11weeks	23	23	30	32	36

* NPD data, so a bit different.

Table S9. Estimated mole fraction of M in % for Al doped Ni35 from XRPD data. The estimations are approximate, as the compositions of M and R are uncertain.

	Al00	Al01	Al03	Al05	Al10
Ref [2]	48	76	56	63	-
14h	58	65	50	54	-
43h	55	52	69	56	-
1week	44	49	59	64	-
3weeks	44	49	58	60	63
11weeks	42	42	43	46	44

* NPD data.

S3.2.2. ^{7}Li MAS NMR on tempered Ni35-Al samples

^{7}Li MAS NMR experiments were performed at the magnetic field strength of 14.1 T (Larmor frequency 233.23 MHz) with a Bruker Avance-III NMR spectrometer equipped with a 1.3 mm MAS probehead. The 60.00 kHz MAS rate was employed. Acquisitions involved a rotor-synchronized, double-adiabatic spin-echo sequence with a 90° excitation pulse of 1.00 µs followed by a pair of 50.0 µs tanh/tan short, high-power adiabatic pulses (SHAPs) with a 5 MHz frequency sweep. All pulses operated at the nutation frequency of 250 kHz. 4096 signal transients with 0.5 s relaxation delay were collected. NMR shifts were referenced with respect to solid LiF.

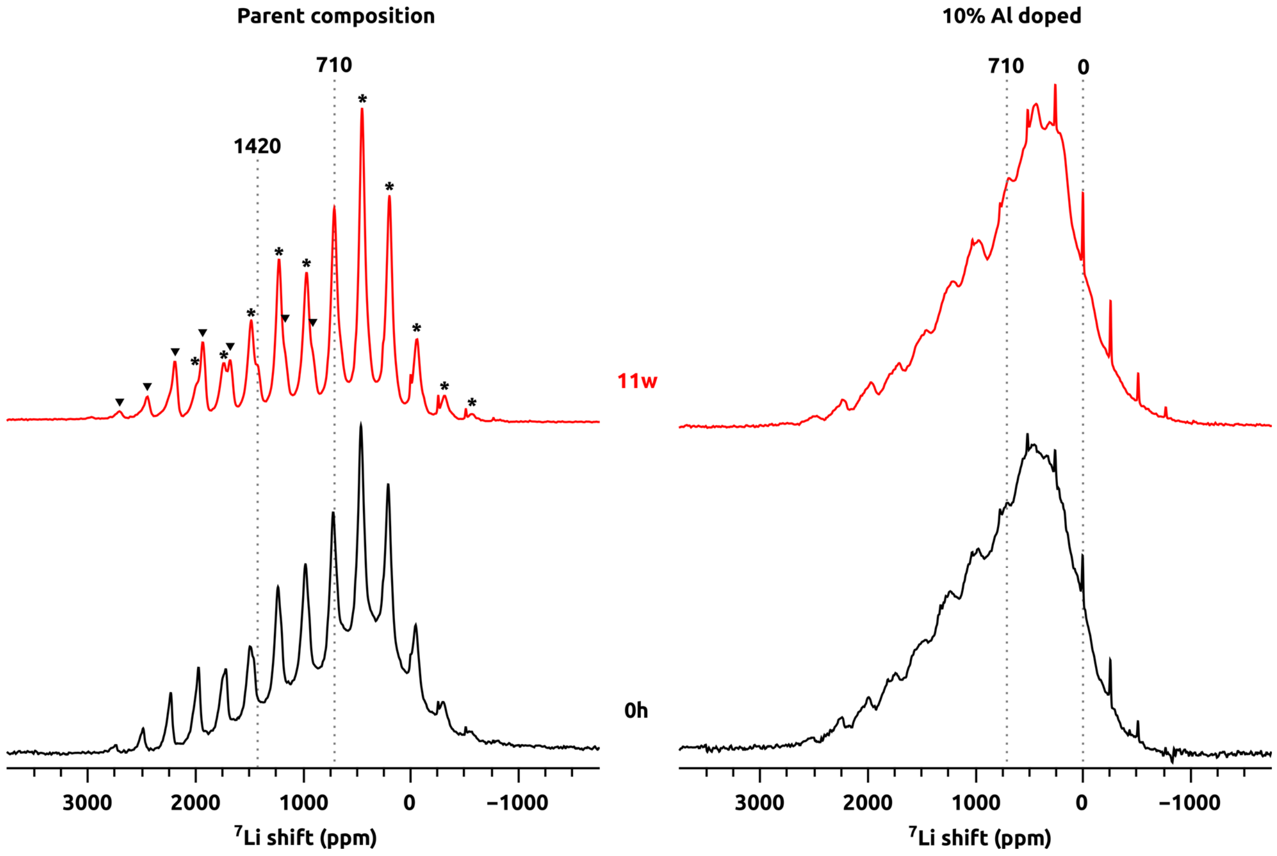

Figure S7. ^{7}Li MAS NMR spectra of undoped (left panel) and 10% Al doped specimens collected for as synthesized (black traces) and heat treated samples for 11 weeks (red traces).

S3.2.3. The degree of size and strain sample broadening.

The sample broadening is dominated by strain. The first peak is relatively sharp and peaks become markedly broader as 2θ increases. Below is a comparison between the actual calculated pattern and a pattern calculated with no sample broadening for Al05 t = 43h for two different 2θ regions showing an increased broadening at higher scattering angles,

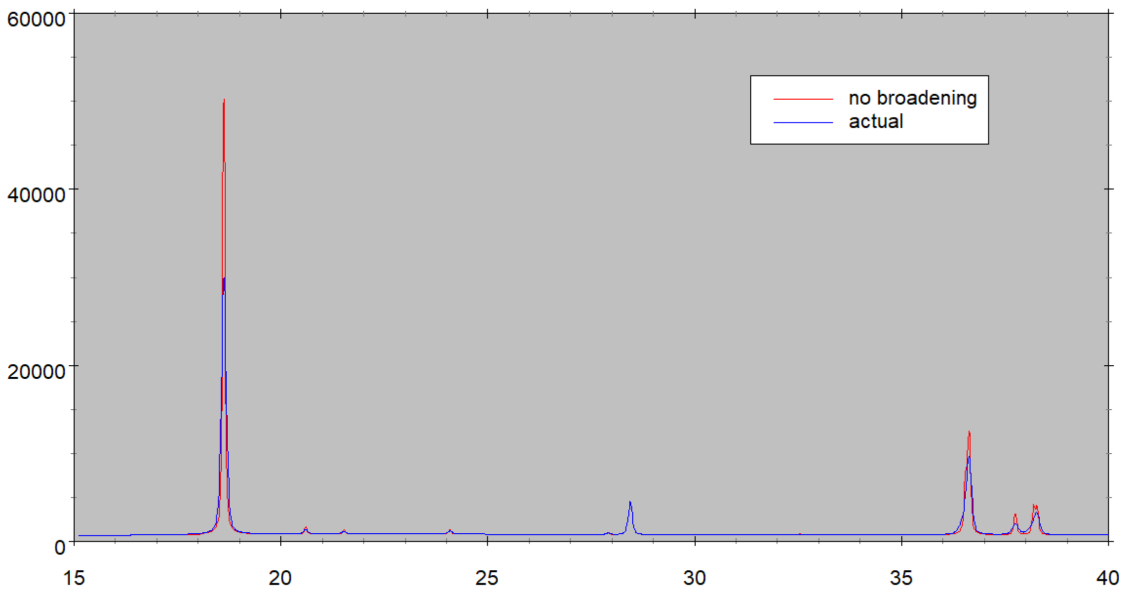

Figure S8. Calculated actual (blue) and with no sample broadening (red) powder patterns (15° ≤ 2θ ≤ 40) for Ni35-Al01 heat treated for 3 weeks. Note that the first peak is only marginally broadened.

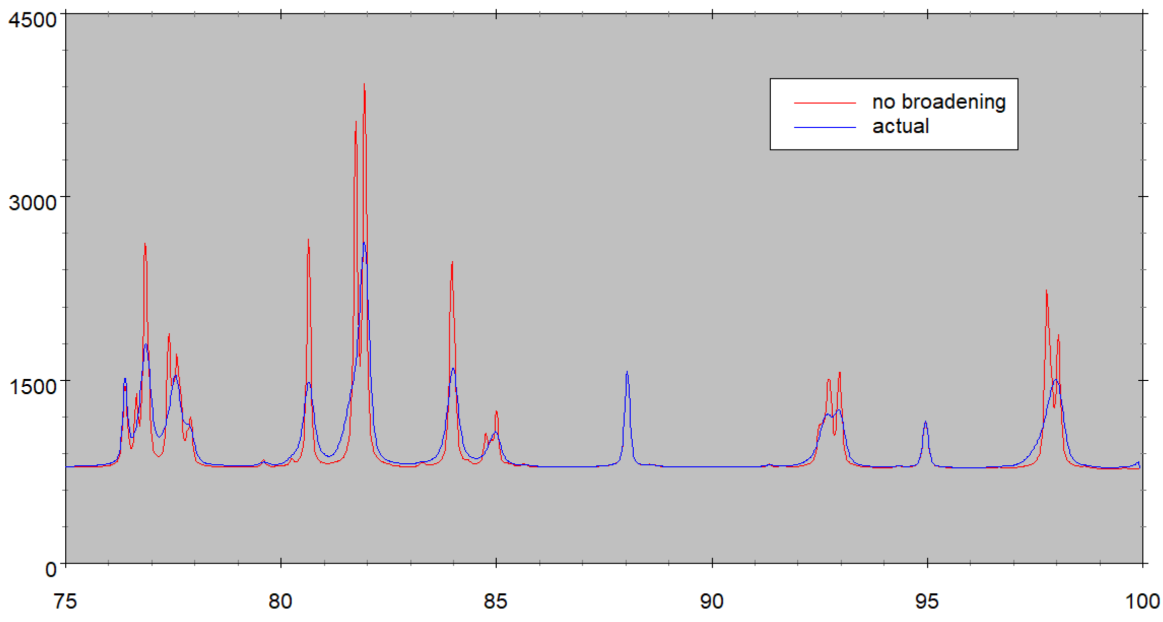

Figure S9. Calculated actual (blue) and with no sample broadening (red) powder patterns (75° ≤ 2θ ≤ 100°) for Ni35- Al01 heat treated for 3 weeks.

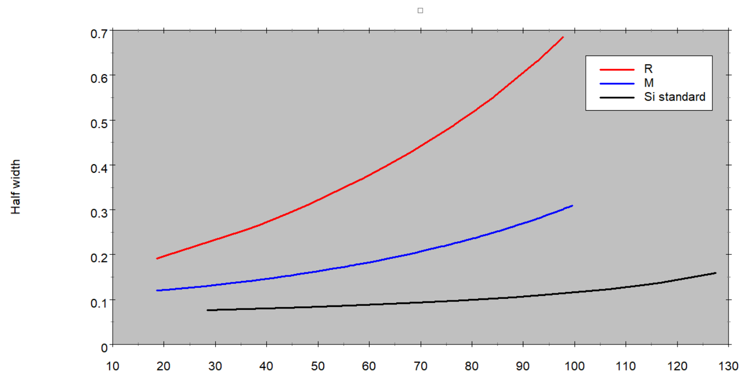

Figure S10. Peak half-widths as a function of 2θ for M (blue) and R(red) for Ni35-Al01 heat treated for 3 weeks. The instrumental broadening was estimated from the halfwidths for the NIST 640c Si standard (black).

S3.2.4. Size and strain parameters for long heat treatments *of Ni35-Al.*

Table S10. Size and strain for M and R for long term heat treatments of Ni35-Al. Shaded rows are not plotted in Figure 8.

sample	Size (Å)	Error	Strain·10^4	Error
0.00 14h M	1083	57	14.4	0.7
0.00 43h M	1130	88	15.5	1.0
0.00 1w M**	**	**	26.8	1.0
0.00 3w M**	**	**	23.2	1.0
0.00 11w M**,***	**	**	23.6	1.5
0.01 14h M	978	44	14.5	0.7
0.01 43h M	1112	68	12.9	0.7
0.01 1w M**	**	**	21.2	0.7
0.01 3w M**	**	**	21.0	0.7
0.01 11w M**,***	**	**	**	**
0.03 14h M	1490	121	13.2	0.7
0.03 43h M	995	37	12.8	0.6
0.03 1w M	1953	323	14.9	0.8
0.03 3w M	2495	736	16.0	0.9
0.03 11w M**,***	**	**	22.0	0.9
0.05 14h M	1127	59	13.6	0.7
0.05 43h M	1387	92	10.8	0.7
0.05 1w M	1566	122	9.8	0.7
0.05 3w M	1677	174	10.1	0.8
0.05 11w M**	**	**	14.0	0.7
0.10 14h M*	676	130	17.7	0.5
0.10 43h M*	764	151	14.9	0.4
0.10 1w M*	984	327	11.3	0.6
0.10 3w M	1592	1103	8.8	0.6
0.10 11w M**	**	**	10.5	0.9
0.00 14h R	1339	115	10.0	1.1
0.00 43h R	1747	228	10.5	1.1

0.00 1w R	2844	1104	22.5	1.1
0.00 3w R**	**	**	19.6	0.7
0.00 11w R**,***	**	**	**	**
0.01 14h R	737	38	10.3	1.6
0.01 43h R	1393	121	8.3	1.1
0.01 1w R	1928	306	14.4	1.1
0.01 3w R	2821	803	13.0	1.0
0.01 11w R**,***	**	**	20.0	2.2
0.03 14h R	416	11	13.8	1.6
0.03 43h R	540	26	11.4	2.3
0.03 1w R	1634	205	10.1	1.2
0.03 3w R	2098	379	9.3	1.1
0.03 11w R***	2824	1094	15.6	2.3
0.05 14h R	419	7	12.6	1.4
0.05 43h R	492	19	15.3	1.8
0.05 1w R	1463	164	9.9	1.2
0.05 3w R	1748	262	10.8	1.2
0.05 11w R**	**	**	19.3	0.7
0.10 14h R*	676	130	17.7	0.5
0.10 43h R*	764	151	14.9	0.4
0.10 1w R*	984	33	11.3	0.6
0.10 3w R	1013	70	10.8	1.3
0.10 11w R**	**	**	12.9	0.9

* Common parameters for M and R

** Instrumental resolution limited = no size broadening

*** Asymmetric size broadening for R

S3.3. Ni15 M phases.

Table S11. Unit cell parameters for Ni15 M phases from PXRD data. Statistical errors are ~0.003 Å, ~0.005 Å, ~0.003 Å and ~0.02° for *a*, *b*, *c* and β.

Sample	*a* (Å)	*b* (Å)	*c* (Å)	β (°)	V* (Å^3)
Al00	4.950	8.574	5.025	109.08	33.59
Al01	4.950	8.569	5.027	109.10	33.58
1week 900°C	4.955	8.578	5.035	109.28	33.67
24h 1000°C	4.954	8.581	5.030	109.05	33.68
Al03	4.949	8.567	5.022	109.05	33.55
Al05	4.944	8.564	5.022	109.03	33.50
**	4.944	8.564	5.021	109.02	33.50
Al10	4.938	8.554	5.020	108.98	33.41

* Normalised.

** A bigger batch

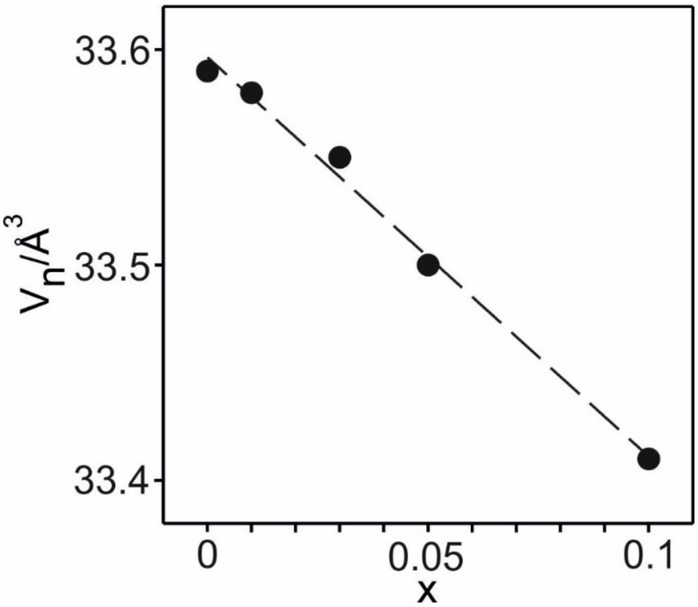

Fig. S11. Unit cell volume V *vs. x* for Ni15 M phases from PXRD.

Table S12. Strain and size for Ni15 M phases.

Sample	Size M (Å)	Strain·10^4 M
Al00	1113	24
Al01	815	18
1week 900°C	2097	8
24h 1000°C	*	14
Al03	609	16
Al05	1094	17
Al10	1247	19

* Instrumental resolution limited = no significant broadening.

S4. Neutron powder diffraction (NPD)
S4.1. Refinements

The neutron scattering lengths used are for Mn -0.373, for Ni 1.03, for Li -0.19, for Al 0.345 and for O 0.580 10^{-12} cm. Three sets of refinements were made;

A. The nominal compositions were used (with the Li content decreased to 1.24 to give an equal number of cations and anions). Mn, Al, Ni and Li were put on the 4g site and only Li on the 2b site. Ni and Li was then allowed to transfer between the 4g and 2b sites. The 4h and 2c sites do only contain Li.
B. The nominal compositions were used as in A. The 4g sites were assumed to contain only Mn, Al and Ni and the 2b sites by the remaining Ni and Li. The site occupancies were here thus fixed. The 4h and 2c sites do only contain Li.
C. The starting occupations were as in B. The composition was then refined by allowing it to vary according to $3Ni^{2+} \leftrightarrow 2Li^{+} + 1Mn^{4+}$ (keeping a charge balance) and assuming only Mn, Al and Ni on the 4g sites. The 4h and 2c sites do only contain Li.

Approximately 25 parameters were used in the final refinements. They included for M 7 atom position parameters, a common temperature (thermal displacement) factor for atoms on 4g and 2b sites, a common temperature factor for Li3 and Li4 on 2c and 4h sites, a common temperature factor for O1 and O2, 4 unit cell parameters. A pseudo-Voigt function (no. 5) was used for peak shapes and U, V, W and shape (η) refined. Li_2CO_3 was included as a secondary phase. The high $\chi 2$ values can be attributed mainly to good intensity statistics.

The refinements of set A showed that Ni is predominantly on site 2b and that 4g contains no significant amount of Li for x 0.05 and 0.10, only Mn, Al and Ni. (The refined site occupancies of Li on 4g is actually slightly negative.) The residuals for refinements of sets B (fixed nominal composition) and C (composition refined) are very similar. We conclude that the NPD data can be fitted well with assuming the nominal compositions (set B).

S4.2. Results

Table S13. Compositions of 4g and 2b sites for refinement sets A, B and C.

sample	Refinement set	4g	2b	wt% Li_2CO_3
Al00	A	$Mn_{3.66}Ni_{0.53(5)}Li_{-0.20(5)}$	$Li_{1.63(5)}Ni_{0.37(5)}$	
	B	$Mn_{3.66}Ni_{0.34}$	$Li_{1.44}Ni_{0.56}$	1.0(1)
	C	$Mn_{3.70(5)}Ni_{0.30(5)}$	$Li_{1.53(5)}Ni_{0.47(5)}$	
Al05	A	$Mn_{3.36}Al_{0.30}Ni_{0.43(5)}Li_{-0.09(5)}$	$Li_{1.53(5)}Ni_{0.47(5)}$	
	B	$Mn_{3.36}Al_{0.30}Ni_{0.34}$	$Li_{1.44}Ni_{0.56}$	1.1(1)
	C	$Mn_{3.37(5)}Al_{0.30}Ni_{0.33(5)}$	$Li_{1.47(5)}Ni_{0.53(5)}$	
Al10	A	$Mn_{3.06}Al_{0.60}Ni_{0.42(5)}Li_{-0.08(5)}$	$Li_{1.52(5)}Ni_{0.48(5)}$	
	B	$Mn_{3.06}Al_{0.60}Ni_{0.34}$	$Li_{1.44}Ni_{0.56}$	2.4(1)
	C	$Mn_{3.09(5)}Al_{0.60}Ni_{0.32(5)}$	$Li_{1.49(5)}Ni_{0.51(5)}$	

* nominal composition, **refined using $3Ni^{2+} \leftrightarrow 2Li^{+} + 1Mn^{4+}$.

Table S14. Reliability indices for refinements for Ni15 M phases using NPD data.

	Refinement set	$\chi2$	R_F(M) (%)	Composition
Al00	A	26	4.3	$Li_{1.24}Mn_{0.61}Ni_{0.15}O_2$*
	B	33	4.8	$Li_{1.24}Mn_{0.61}Ni_{0.15}O_2$*
	C	29	4.7	$Li_{1.25}Mn_{0.62}Ni_{0.13}O_2$**
Al05	A	31	4.5	$Li_{1.24}Mn_{0.56}Al_{0.05}Ni_{0.15}O_2$*
	B	33	4.8	$Li_{1.24}Mn_{0.56}Al_{0.05}Ni_{0.15}O_2$*
	C	32	4.9	$Li_{1.24}Mn_{0.56}Al_{0.05}Ni_{0.14}O_2$**
Al10	A	31	4.7	$Li_{1.24}Mn_{0.51}Al_{0.10}Ni_{0.15}O_2$*
	B	32	4.8	$Li_{1.24}Mn_{0.51}Al_{0.10}Ni_{0.15}O_2$*
	C	30	4.9	$Li_{1.25}Mn_{0.51}Al_{0.10}Ni_{0.14}O_2$**

* nominal composition, **refined using $3Ni^{2+} \leftrightarrow 2Li^{+} + 1Mn^{4+}$.

Table S15. Unit cell parameters for Ni15 M phases from NPD data (data set B).

sample	*a* (Å)	*b* (Å)	*c* (Å)	β (°)	V (Å^3)	Vn (Å^3)
Al00	4.9578(5)	8.5774(9)	5.0354(4)	109.269(9)	202.13	33.69
Al05	4.9531(4)	8.5709(7)	5.0355(3)	109.300(6)	201.76	33.63
Al10	4.9443(4)	8.5646(6)	5.0252(2)	108.985(6)	201.22	33.53
[2]*	4.9506(10)	8.5944(15)	5.0375(9)	109.44(2)	202.11	33.69

* Ni35-Al00, NPD data.

Table S16. Atomic coordinates for Al doped Ni15 M phases from NPD data (data set B).

sample	x-O2	y-O2	z-O2	y-Mn	y-Li4	x-O1	z-O1
Al00	0.246(3)	0.326(1)	0.226(1)	0.168(4)	0.661(6)	0.221(3)	0.228(3)
Al05	0.250(3)	0.326(1)	0.224(1)	0.165(4)	0.658(5)	0.224(4)	0.227(3)
Al10	0.248(3)	0.324(1)	0.224(1)	0.168(5)	0.658(5)	0.224(3)	0.225(3)
[2]*	0.2631(11)	0.3187(4)	0.2192(7)	0.1616(9)	0.6921(13)	0.2185(14)	0.2292(11)

* Ni35-Al00, NPD data.

Table S17. Metal-O distances in the transition metal layer for Ni15 M phases from NPD data (data set B). Calculated values are calculated using refined site occupancies and Shannon-Prewitt radii; Li^+ = 0.76 Å, Ni^{2+} = 0.690 Å, Ni^{3+} = 0.60 Å, Mn^{3+} = 0.645 Å, Mn^{4+} = 0.53 Å, Al^{3+} = 0.535 Å, O^{2-} = 1.40 Å.

sample	4g-O2	4g-O2	4g-O1	Mean	calc	2b-O2	2b-O1	mean	calc
Al00	1.954(13)	1.925(30)	1.938(30)	1.939	1.952	2.025(10)	2.068(17)	2.039	2.140
Al05	1.932(13)	1.946(30)	1.922(30)	1.933	1.952	2.027(9)	2.049(20)	2.036	2.140
Al10	1.935(10)	1.911(30)	1.940(30)	1.929	1.952	2.035(9)	2.038(17)	2.036	2.140
[2]*	1.945(7)	1.864(5)	1.897(5)	1.902	1.943	2.094(4)	2.082(7)	2.090	2.127

* Ni35-Al00.

Table S18. Li-O distances in the Li metal layer for Al doped M phases.

sample	Li3-O2	Li3-O1	Mean	Calc	Li4-O2	Li4-O2	Li4-O1	Mean	calc
Al00	2.140(9)	2.018(17)	2.099	2.160	2.127(13)	2.083(4)	2.109(3)	2.107	2.160
Al05	2.135(8)	2.034(20)	2.101	2.160	2.151(13)	2.094(30)	2.087(30)	2.111	2.160
Al10	2.151(9)	2.031(17)	2.111	2.160	2.133(13)	2.091(39)	2.093(30)	2.106	2.160
[2]*	2.167(3)	1.999(7)	2.111	2.160	2.217(5)	1.861(7)	2.294(9)	2.124	2.160

* Ni35-Al00.

Table S19. Bond valence sums (BVS) for O atoms. Used d_0 values; Li^{1+} 1.446, Mn^{4+} 1.753, Ni^{2+} 1.654, Al^{3+} 1.620.

Sample	O2	O1
Al00	1.93	1.96
Al05	1.89	2.01
Al10	1.91	1.97
[2]*	2.14	2.00

* Ni35-Al00.

S5. Miscellaneous

S5.1. Dependence on unit cell volume by mean Shannon-Prewitt cation radius.

The normalised unit cell volume *V* depends on the mean Shannon-Prewitt cation radius r as ~V ∝ 115.1*r.

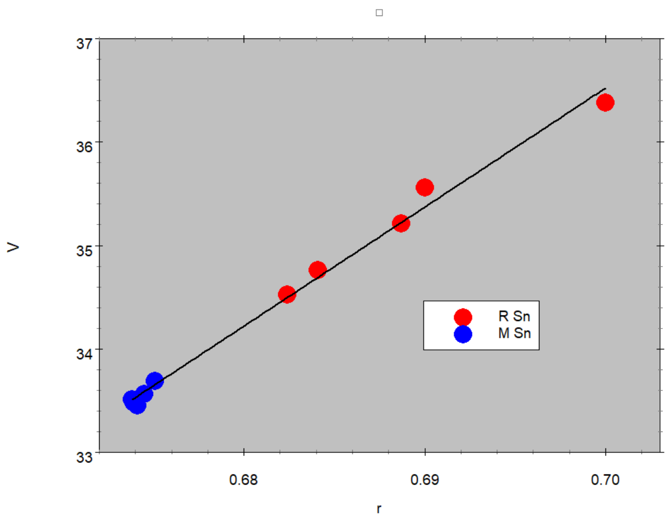

Figure S12. Unit cell volume V *vs.* the mean Shannon-Prewitt of cations in M and R phases (data from [2]).

An increase of r by 0.001 Å corresponds to an increase of V by 0.12 $Å^3$. This shows that very small changes in the cation composition is needed to give significant changes in V.

E.g., assume that initially a charge balance is realised for Ni35 Al x 0.10 by forming an amount of Ni^{3+} that equals the amount of Al^{3+} $Li_{1.1}Mn_{0.45}Al_{0.10}Ni^{2+}_{0.35}O_2 \rightarrow Li_{1.1}Mn_{0.45}Al_{0.10}Ni^{2+}_{0.25}Ni^{3+}_{0.10}O_2$. The charge balancing decreases r formally by 0.0045 Å, corresponding to a decrease in V by 0.52 $Å^3$. More detailed calculations like this are of course not very meaningful.
